# Lactate is associated with mortality in very old intensive care patients suffering from COVID-19: results from an international observational study of 2860 patients

**DOI:** 10.1186/s13613-021-00911-8

**Published:** 2021-08-21

**Authors:** Raphael Romano Bruno, Bernhard Wernly, Hans Flaatten, Jesper Fjølner, Antonio Artigas, Bernardo Bollen Pinto, Joerg C. Schefold, Stephan Binnebössel, Philipp Heinrich Baldia, Malte Kelm, Michael Beil, Sivri Sigal, Peter Vernon van Heerden, Wojciech Szczeklik, Muhammed Elhadi, Michael Joannidis, Sandra Oeyen, Tilemachos Zafeiridis, Jakob Wollborn, Maria José Arche Banzo, Kristina Fuest, Brian Marsh, Finn H. Andersen, Rui Moreno, Susannah Leaver, Ariane Boumendil, Dylan W. De Lange, Bertrand Guidet, Christian Jung, the COVIP Study Group, Philipp Eller, Michael Joannidis, Dieter Mesotten, Pascal Reper, Sandra Oeyen, Walter Swinnen, Nicolas Serck, Elisabeth Dewaele, Edwin Chapeta, Helene Brix, Jens Brushoej, Pritpal Kumar, Helene Korvenius Nedergaard, Tim Koch Johnsen, Camilla Bundesen, Maria Aagaard Hansen, Stine Uhrenholt, Helle Bundgaard, Jesper Fjølner, Richard Innes, James Gooch, Lenka Cagova, Elizabeth Potter, Michael Reay, Miriam Davey, Mohammed Abdelshafy Abusayed, Sally Humphreys, Amy Collins, Avinash Aujayeb, Susannah Leaver, Waqas Khaliq, Ayman Abdelmawgoad Habib, Mohammed A. Azab, Kyrillos Wassim, Yumna A. Elgazzar, Rehab Salah, Hazem Maarouf Abosheaishaa, Aliae A. R. Hussein Mohamed, Ahmed Y. Azzam, Samar Tharwat, Yasmin Khairy Nasreldin Mohamed Ali, Omar Elmandouh, Islam Galal, Ahmed Abu-Elfatth, Karam Motawea, Mohammad Elbahnasawy, Mostafa Shehata, Mohamed Elbahnasawy, Mostafa Tayeb, Nermin Osman, Wafaa Abdel-Elsalam, Aliae Mohamed Hussein, Amer Aldhalia, Arnaud Galbois, Bertrand Guidet, Cyril Charron, Caroline Hauw Berlemont, Guillaume Besch, Jean-Philippe Rigaud, Julien Maizel, Michel Djibré, Philippe Burtin, Pierre Garcon, Saad Nseir, Xavier Valette, Nica Alexandru, Nathalie Marin, Marie Vaissiere, Gaëtan Plantefeve, Hervé Mentec, Thierry Vanderlinden, Igor Jurcisin, Buno Megarbane, Benjamin Glenn Chousterman, François Dépret, Marc Garnier, Sebastien Besset, Johanna Oziel, Alexis Ferre, Stéphane Dauger, Guillaume Dumas, Bruno Goncalves, Lucie Vettoretti, Didier Thevenin, Stefan Schaller, Muhammed Kurt, Andreas Faltlhauser, Christian Meyer, Milena Milovanovic, Matthias Lutz, Gonxhe Shala, Hendrik Haake, Winfried Randerath, Anselm Kunstein, Patrick Meybohm, Stephan Steiner, Eberhard Barth, Tudor Poerner, Philipp Simon, Marco Lorenz, Zouhir Dindane, Karl Friedrich Kuhn, Martin Welte, Ingo Voigt, Hans-Joachim Kabitz, Jakob Wollborn, Ulrich Goebel, Sandra Emily Stoll, Detlef Kindgen-Milles, Simon Dubler, Christian Jung, Kristina Fuest, Michael Schuster, Stephan Steiner, Antonios Papadogoulas, Francesk Mulita, Nikoletta Rovina, Zoi Aidoni, Evangelia Chrisanthopoulou, Eumorfia Kondili, Ioannis Andrianopoulos, Mohan Gurjar, Ata Mahmoodpoor, Rand Hussein, Maytham Aqeel Al-Juaifari, Abdullah Khudhur Ahmed Karantenachy, Sigal Sviri, Ahmed Elsaka, Brian Marsh, Vittoria Comellini, Farah Al-Ali, Sari Almani, Almu’ Atasim Khamees, Khayry Al-Shami, Ibrahim Salah El Din, Taha Abubaker, Hazem Ahmed, Ahmed Rabha, Abdulmueti Alhadi, Marwa Emhamed, Saedah Abdeewi, Abdurraouf Abusalama, Abdulmueti Alhadi, Mohammed Huwaysh, Esraa Abdalqader Alghati, Abdelilah Ghannam, Silvio A. Namendys-Sylva, Martijn Groenendijk, Mirjam Evers, Lenneke Van Lelyveld-Haas, Iwan Meynaar, Alexander Daniel Cornet, Marieke Zegers, Willem Dieperink, Dylan De Lange, Tom Dormans, Michael Hahn, Britt Sjøbøe, Hans Frank Strietzel, Theresa Olasveengen, Luis Romundstad, Finn H. Andersen, John George Grace Massoud, Aamir Ghafoor Khan, Shahd Al-Qasrawi, Sarah Amro, Anna Kluzik, Paweł Zatorski, Tomasz Drygalski, Wojciech Szczeklik, Jakub Klimkiewicz, Joanna Solek-Pastuszka, Dariusz Onichimowski, Miroslaw Czuczwar, Ryszard Gawda, Jan Stefaniak, Karina Stefanska-Wronka, Ewa Zabul, Ana Isabel Pinho Oliveira, Rui Assis, Maria De Lurdes Campos Santos, Henrique Santos, Filipe Sousa Cardoso, André Gordinho, Ioana Marina Grintescu, Dana Tomescu, Mohamed Raafat Badawy, M. José Arche Banzo, Begoña Zalba-Etayo, Patricia Jimeno Cubero, Jesús Priego, Gemma Gomà, Teresa Maria Tomasa-Irriguible, Susana Sancho, Aida Fernández Ferreira, Eric Mayor Vázquez, Ángela Prado Mira, Mercedes Ibarz, David Iglesias, Susana Arias-Rivera, Fernando Frutos-Vivar, Sonia Lopez-Cuenca, Cesar Aldecoa, David Perez-Torres, Isabel Canas-Perez, Luis Tamayo-Lomas, Cristina Diaz-Rodriguez, Pablo Ruiz De Gopegui, Mahmoud Saleh, Momin Majed Yousuf Hilles, Enas M. Y. Abualqumboz, Nawfel Ben-Hamouda, Andrea Roberti, Yvan Fleury, Nour Abidi, Joerg C. Schefold, Ivan Chau, Alexander Dullenkopf, Mohammad Karam Chaaban, Mohammed Mouaz Shebani, Ahmad Hmaideh, Aymen Shaher, Ayca Sultan Sahin, Kemal Tolga Saracoglu, Mohammed Al-Sadawi, Richard Pugh, Sara Smuts, Rafat Ameen Mohammed Al-Saban

**Affiliations:** 1 Department of Cardiology, Pulmonology and Vascular Medicine, Medical Faculty, Heinrich-Heine-University Duesseldorf, Moorenstraße 5, 40225 Duesseldorf, Germany; 2 Department of Anaesthesiology, Perioperative Medicine and Intensive Care Medicine, Paracelsus Medical University, Salzburg, Austria; 3 Department of Clinical Medicine, University of Bergen, Bergen, Norway; 4 Department of Anaestesia and Intensive Care, Haukeland University Hospital, Bergen, Norway; 5 Department of Intensive Care, Aarhus University Hospital, Aarhus, Denmark; 6 Department of Intensive Care Medicine, CIBER Enfermedades Respiratorias, Corporacion Sanitaria Universitaria Parc Tauli, Autonomous University of Barcelona, Sabadell, Spain; 7 Department of Acute Medicine, Geneva University Hospitals, Geneva, Switzerland; 8 Department of Intensive Care Medicine, Inselspital, Universitätsspital, University of Bern, Bern, Switzerland; 9 Deptartment of Medical Intensive Care, Hadassah Medical Center and Faculty of Medicine, Hebrew University of Jerusalem, Jerusalem, Israel; 10 General Intensive Care Unit, Deptartment of Anesthesiology, Critical Care and Pain Medicine, Hadassah Medical Center and Faculty of Medicine, Hebrew University of Jerusalem, Jerusalem, Israel; 11 Center for Intensive Care and Perioperative Medicine, Jagiellonian University Medical College, Krakow, Poland; 12 Faculty of Medicine, University of Tripoli, Tripoli, Libya; 13 Division of Intensive Care and Emergency Medicine, Department of Internal Medicine, Medical University Innsbruck, Innsbruck, Austria; 14 Department of Intensive Care 1K12IC, Ghent University Hospital, Ghent, Belgium; 15 Intensive Care Unit General Hospital of Larissa, Larissa, Greece; 16 Department of Anesthesiolgy, Perioperative and Pain Medicine, Brigham and Women’s Hospital, Harvard Medical School, Boston, USA; 17 Hospital Clínico Universitario Lozano Blesa, Zaragoza, Spain; 18 Department of Anesthesiology and Intensive Care, Klinikum Rechts Der Isar, Technical University of Munich, Munich, Germany; 19 Mater Misericordiae University Hospital, Dublin, Ireland; 20 Department of Anaesthesia and Intensive Care, Ålesund Hospital, Ålesund, Norway; 21 Department of Circulation and Medical Imaging, Norwegian University of Science and Technology, Trondheim, Norway; 22 Unidade de Cuidados Intensivos Neurocríticos E Trauma, Hospital de São José, Centro Hospitalar Universitário de Lisboa Central, Faculdade de Ciências Médicas de Lisboa, Nova Medical School, Lisbon, Portugal; 23 General Intensive Care, St George´S University Hospitals NHS Foundation Trust, London, UK; 24 Institut Pierre Louis D’Epidémiologie Et de Santé Publique, Equipe: épidémiologie hospitalière qualité et organisation des soins, Sorbonne Universités, UPMC Univ Paris 06, INSERM, UMR_S 1136, 75012 Paris, France; 25 Assistance Publique–Hôpitaux de Paris, service de réanimation médicale, Hôpital Saint-Antoine, 75012 Paris, France; 26 Department of Intensive Care Medicine, University Medical Center, University Utrecht, Utrecht, The Netherlands

## Abstract

**Purpose:**

Lactate is an established prognosticator in critical care. However, there still is insufficient evidence about its role in predicting outcome in COVID-19. This is of particular concern in older patients who have been mostly affected during the initial surge in 2020.

**Methods:**

This prospective international observation study (The COVIP study) recruited patients aged 70 years or older (ClinicalTrials.gov ID: NCT04321265) admitted to an intensive care unit (ICU) with COVID-19 disease from March 2020 to February 2021. In addition to serial lactate values (arterial blood gas analysis), we recorded several parameters, including SOFA score, ICU procedures, limitation of care, ICU- and 3-month mortality. A lactate concentration ≥ 2.0 mmol/L on the day of ICU admission (baseline) was defined as abnormal. The primary outcome was ICU-mortality. The secondary outcomes 30-day and 3-month mortality.

**Results:**

In total, data from 2860 patients were analyzed. In most patients (68%), serum lactate was lower than 2 mmol/L. Elevated baseline serum lactate was associated with significantly higher ICU- and 3-month mortality (53% vs. 43%, and 71% vs. 57%, respectively, *p* < 0.001). In the multivariable analysis, the maximum lactate concentration on day 1 was independently associated with ICU mortality (aOR 1.06 95% CI 1.02–1.11; *p* = 0.007), 30-day mortality (aOR 1.07 95% CI 1.02–1.13; *p* = 0.005) and 3-month mortality (aOR 1.15 95% CI 1.08–1.24; *p* < 0.001) after adjustment for age, gender, SOFA score, and frailty. In 826 patients with baseline lactate ≥ 2 mmol/L sufficient data to calculate the difference between maximal levels on days 1 and 2 (∆ serum lactate) were available. A decreasing lactate concentration over time was inversely associated with ICU mortality after multivariate adjustment for SOFA score, age, Clinical Frailty Scale, and gender (aOR 0.60 95% CI 0.42–0.85; *p* = 0.004).

**Conclusion:**

In critically ill old intensive care patients suffering from COVID-19, lactate and its kinetics are valuable tools for outcome prediction.

*Trial registration number*: NCT04321265.

**Supplementary Information:**

The online version contains supplementary material available at 10.1186/s13613-021-00911-8.

## Introduction

The disease caused by Sars-CoV-2, COVID-19, has dominated daily life in numerous intensive care units (ICU) worldwide, since the beginning of 2020. Respiratory failure with or without shock led to high mortality. In ICU admitted patients, up to 30–50% of the patients did not survive the first month [1–3]. Thus, early and reliable identification of complex disease courses is of pivotal importance in COVID-19 (Additional file 1).

In emergency and critical care medicine, serum lactate and its kinetics are useful parameters for critically ill patients as a marker of severity of illness [4–6]. A significant advantage is that the determination of serum lactate is widely and rapidly available as a point-of-care measurement [7]. Hyperlactatemia is an indicator of physiological stress, and anaerobic metabolism, and a “powerful predictor of mortality” [6]. Basically, lactate can be used for two purposes. It can be used both for risk stratification and to monitor the response to therapy. Elevated lactate is a diagnostic criterion for septic shock following the sepsis-3 consensus. Lactate “clearance” is a target parameter for volume substitution in the absence of major liver dysfunction [8]. Although serum lactate and its kinetics have been applied as an essential diagnostic and target parameter in septic patients for more than 20 years, the evidence remains scarce for patients suffering from pneumonia and ARDS.

Despite this lack of evidence, current guidelines recommend the use of lactate and lactate kinetics in COVID-19 [9]. Until now, the value of serum lactate and its kinetics in predicting a severe course in COVID-19 is unclear. This lack of evidence is especially true in the particularly vulnerable population of very old intensive care unit patients. Yet, this subgroup has been disproportionally affected by the need for ICU admissions and a high mortality [3, 10, 11].

This multicenter study addresses this lack of evidence and investigates the value of serum lactate at admission and its kinetics for outcome prediction in a large prospectively enrolled population of older ICU patients.

## Methods

### Design and settings

This multicenter study is part of the Very old Intensive care Patients (VIP) project and has been endorsed by the European Society of Intensive Care Medicine (ESICM) (https://www.vipstudy.org). The study was registered at ClinicalTrials.gov (ID: NCT04321265) and adhered to the European Union General Data Privacy Regulation (GDPR) directive. This investigation aimed to understand which factors can predict mortality in elderly COVID-19 patients to help detect these patients early (the COVIP study, COVID-19 in very old intensive care patients). As in the previous VIP studies [3, 12, 13], national coordinators recruited the intensive care units (ICUs), coordinated national and local ethical permissions, and supervised patient recruitment at the national level. Ethical approval was mandatory for study participation. In most countries informed consent was obligatory for inclusion. This study extracted patient data from 151 ICUs from 26 independent countries, including European ICUs, and the Asian, African, and Americas.

### Study population

The COVIP study recruited consecutieve patients with proven COVID-19 aged 70 years or older who were admitted to an ICU. The data set was extracted from the COVIP study database on 4th February and contained patients from 19th March 2020 to 4th February 2021. Data collection started at ICU admission. Data about pre-ICU triage were not available. The admission day was defined as day 1, and all consecutive days were numbered sequentially from that date.

### Data collection

All centers used a uniform online electronic case report form (eCRF). Only patients with a documented highest serum lactate value on days 1 and 2 were included for this subgroup analysis (above 2 mmol/L). Reporting was possible both in [mg/dL] or [mmol/L], depending on local routine. For ease of comparison, all laboratory values were converted to [mmol/] (1 mg/dL = 0.111 mmol/L). The first arterial blood gas (ABG) analysis, including pO_2_ [mmHg] and the F_i_O_2_ [%], was recorded to calculate the pO_2_/FiO_2_-ratio on admission. For the sequential organ failure assessment (SOFA) score on admission, each element was entered and the eCRF calculated the total score. Furthermore, we assessed the need for non-invasive or invasive ventilation with its duration, prone positioning, tracheostomy, vasopressor use and renal replacement therapy. The eCRF also documented any limitation of life-sustaining therapy during the ICU-stay. The frailty level prior to the acute illness and hospital admission was assessed using the Clinical Frailty Scale (CFS) [3, 12, 13]. In addition, the eCRF recorded information about gender, age, length of ICU stay symptom onset, and duration of symptoms before ICU-and hospital admission. Furthermore, the eCRF asked about the presence of preexisting comorbidities.

### Lactate and ∆ Lactate

Patients were clustered according to their lactate concentration on ICU admission. The arbitrary cutoff value was an initial lactate concentration ≥ 2.0 mmol/L. A lactate value below 2 mmol/L was defined as within the normal range. ∆ Lactate in the first 24 h was defined as maximum serum lactate at admission minus maximum serum lactate on day 2, divided by lactate at admission multiplied by 100 [4]. A positive value indicates a fall in serum lactate and a negative value signifies rising serum lactate. This has been confirmed in larger cohorts as a valuable and simple tool for outcome prediction [4].

$$\Delta {\text{Lactate}} = 100 \times \frac{{{\text{Maximum serum lactate on admission}}\left[ {\frac{{{\text{mmol}}}}{{\text{L}}}} \right] - {\text{Maximum serum lactate on day}}2\left[ {\frac{{{\text{mmol}}}}{{\text{L}}}} \right]}}{{{\text{Maximum serum lactate on admission}}\left[ {\frac{{{\text{mmol}}}}{{\text{L}}}} \right]}}$$

### Data storage

The eCRF and database were hosted on a secure server in Aarhus University, Denmark.

### Statistical analysis

The primary outcome was ICU mortality, secondary outcomes were 30-day and 3-month mortality. Continuous data points were expressed as median and interquartile range. Differences between independent groups were calculated using the MannWhitney *U* test. Categorical data are expressed as numbers (percentage). The Chi-square test was applied to calculate differences between groups. Univariate und multivariable logistic regression analyses were performed to assess associations with baseline serum lactate and mortality. We chose the co-variables for the multivariable model (age, SOFA score, CFS and gender) based on clinical experience and previous literature [4, 5]. Marginal predictive means with respective 95% confidence intervals (CI) were calculated. All tests were two-sided, and a *p* value of < 0.05 was considered statistically significant. Stata 16 was used for all statistical analyses (StataCorp LLC, 4905 Lakeway Drive, College Station, Brownsville, Texas, USA).

## Results

### Study population

**Fig. 1:**
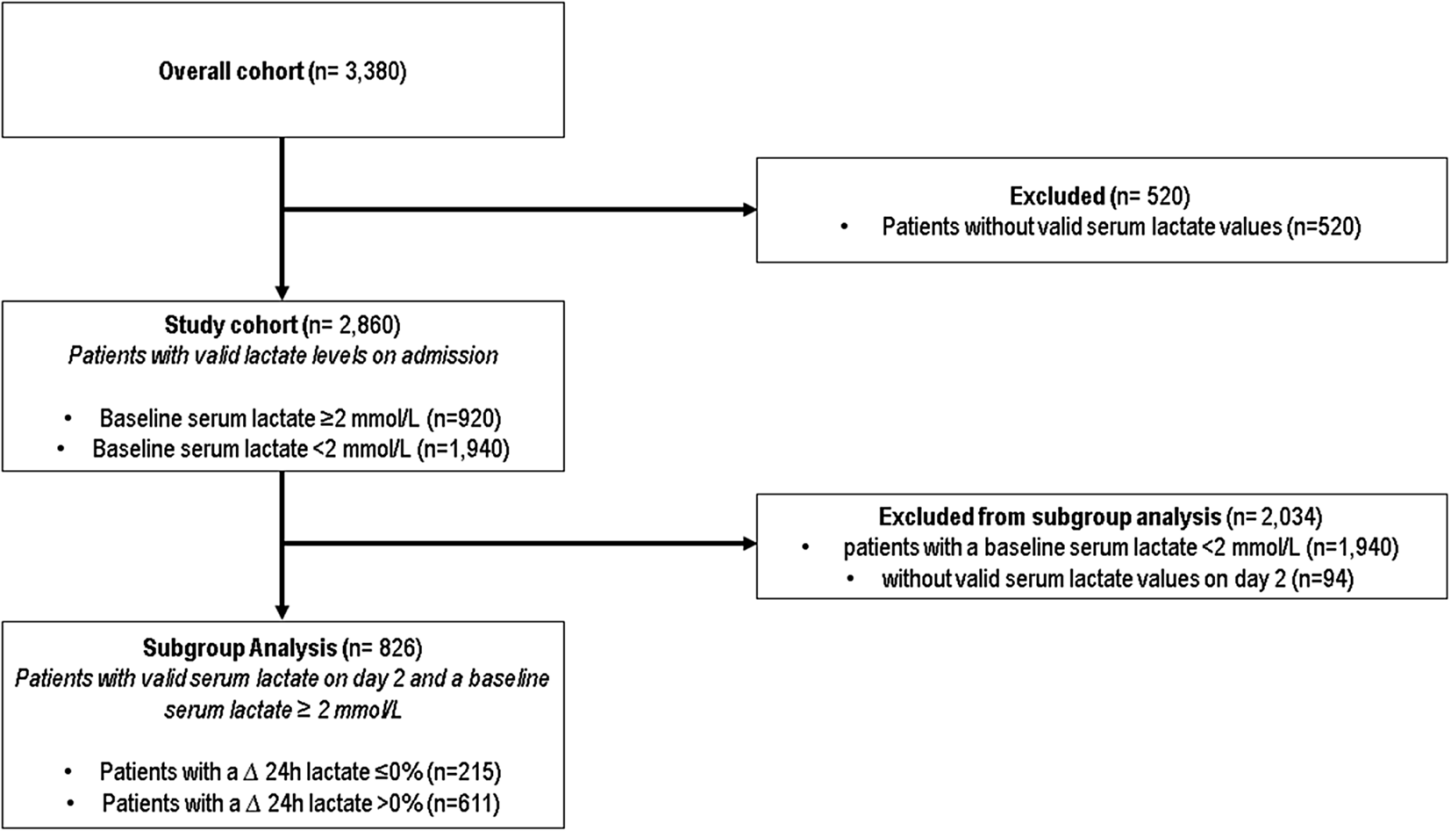
CONSORT diagram

**Fig. 2:**
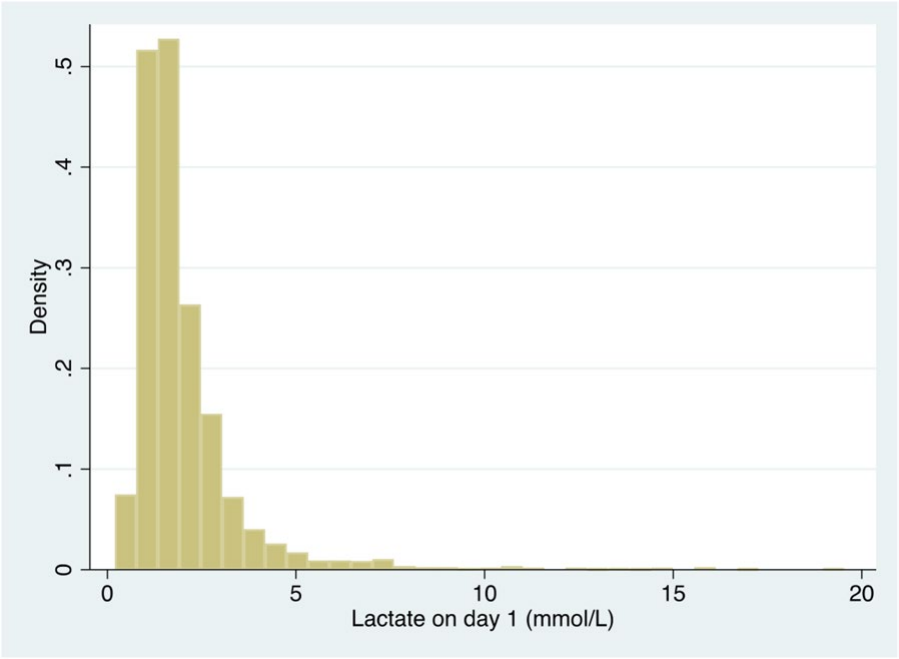
Distribution of maximum serum lactate values on day 1 (= day of ICU-admission), [mmol/L]

In total, 2860 patients with an available baseline serum lactate value were included (Fig. 1). Overall, 68% (1940 patients) patients had no abnormal elevation of serum lactate on the day of admission to the ICU, while 32% (920 patients) evidenced an elevated serum lactate (Fig. 2) (Table 1).

**Table 1:** Baseline characteristics

Variables	Baseline lactate ≥ 2 mmol/L	Baseline lactate < 2 mmol/L	*p* value
	*n* = 920 (32%)	*n* = 1940 (68%)	
Male gender [*n*] (%)	678 (74%)	1356 (70%)	0.033
Age [years] (IQR)	72–79 (76)	72–78 (75)	0.007
Age 70–79 [*n*] (%)	715 (78%)	1554 (80%)	0.15
Age 80–89 [*n*] (%)	194 (21%)	373 (19%)	0.24
Age > 90 [*n*] (%)	10 (1%)	12 (1%)	0.18
CFS (IQR)	2–4 (3)	2–4 (3)	0.47
Diabetes [*n*] (%)	334 (37%)	690 (36%)	0.68
Coronary vascular disease [*n*] (%)	226 (25%)	441 (23%)	0.26
Chronic renal failure [*n*] (%)	163 (18%)	331 (17%)	0.65
Arterial hypertension [*n*] (%)	599 (65%)	1325 (69%)	0.086
Pulmonary disease [*n*] (%)	200 (22%)	456 (24%)	0.30
Heart failure [*n*] (%)	164 (18%)	264 (14%)	0.004
Lactate on day 1 [mmol/L] (IQR)	2.3–3.8 (2.8)	1.1–1.7 (1.4)	< 0.001
SOFA score (IQR)	4–8 (6)	3–8 (5)	< 0.001
Symptoms prior to hospitalization (days)	2 (1–5)	2 (1–5)	0.96
Symptoms prior to ICU-admission (days)	6 (3–9)	7 (3–10)	0.38

*CFS* clinical frailty scale, *SOFA* score sequential organ failure Assessment for the first 24 h, *IQR* interquartile range

### Baseline serum lactate

Patients with a lactate greater than/equal to 2 mmol/L were older [76 (72–79) vs. 75 (72–78) years, *p* = 0.007], but not more frail [CFS 3 (2–4) vs. 3 (2–4), *p* = 0.47]. Patients with elevated serum lactate had a higher SOFA score on admission (Table 1). There were no differences in symptom duration or time in the hospital before ICU admission [Symptoms prior to hospitalization 2 days (1–5) vs. 2 days (1–5), *p* = 0.96; symptoms prior to ICU-admission 6 days (3–9) vs. 7 days (3–10), *p* = 0.38]. Both groups evidenced similar incidences of pre-existing comorbidities (diabetes, coronary vascular disease, chronic renal failure, arterial hypertension, pulmonary disease) with the exception for heart failure, which was significantly more often in patients with an elevated baseline serum lactate (18% vs. 14%, *p* = 0.004).

On day 2, the maximum serum lactate was lower in the group of patients with initially elevated serum lactate than on the day of admission, but still significantly higher than in the group of patients with non-elevated lactate on admission [2.2 mmol/L (1.7–3.0) vs. 1.4 mmol/L (1.1–1.8), *p* < 0.001]. Patients with an elevated baseline serum lactate had significantly higher ∆ lactate in 24 h than patients with a normal baseline lactate [25% (0–45) vs. − 7% (− 33–12), p < 0.001] (Table 1). The two groups demonstrated differences in intensive care therapy. Patients with an elevated baseline lactate were significantly more likely to receive mechanical ventilation [74% (677) vs. 70% (1359), *p* = 0.042], renal replacement therapy [19% (171) vs. 15% (288), *p* = 0.013], and vasoactive drugs [72% (654) vs. 68% (1311), *p* = 0.038], but prone positioning occurred significantly less often in patients with an elevated serum lactate [49% (329) vs. 57% (764), *p* < 0.001]. There was no difference regarding non-invasive ventilation [25% (230) vs. 26% (501), *p* = 0.61] or tracheostomy [19% (173) vs. 19% (364), *p* = 0.95]. Patients with elevated baseline lactate had a significantly higher ICU-, 30-day and 3-month mortality (Table 2). Length of ICU was lower in patients with an elevated baseline serum lactate [336 h (408) vs. 377 (433), *p* = 0.037]. After exclusion of non-survivors, there was no difference between both groups [475 h (526) vs. 570 h (552), *p* = 0.48]. Accordingly, the duration of mechanical ventilation was significantly longer in patients with normal baseline [343 h (360) vs. 367 h (360), *p* = 0.026]. Again, after exclusion of non-survivors and patients without mechanical ventilation, there remained no significant difference between both groups [323 h (414) vs. 311 (445) h, *p* = 0.41]. Treatment was withheld in 30% (271) of the patients with an elevated and in 30% (565) of the patients with a normal baseline lactate (*p* = 0.85). Treatment was withdrawn in 21% (188) and 18% (348), respectively (*p* = 0.12).

**Table 2:** Primary and secondary outcomes

Variables	Baseline Lactate ≥ 2 mmol/L	Baseline Lactate < 2 mmol/L	*p* value
	*n* = 920 (32%)	*n* = 1940 (68%)	
ICU mortality	465 (53%)	817 (43%)	< 0.001
30-day mortality	496 (56%)	854 (46%)	< 0.001
3-month mortality	533 (71%)	924 (57%)	< 0.001
ICU length of stay (hours, IQR)	336 (408)	377 (433)	0.037
Duration of mechanical ventilation (hours, IQR)	343 (360)	367 (360)	0.026

*ICU* intensive care unit

In a univariate regression analysis, the baseline lactate was significantly associated with ICU mortality (OR 1.12 95% CI 1.07–1.17; *p* < 0.001), 30-day mortality (OR 1.11 95% CI 1.06–1.16; *p* < 0.001) and 3-month mortality (OR 1.16 95% CI 1.09–1.23; *p* < 0.001).

**Fig. 3:**
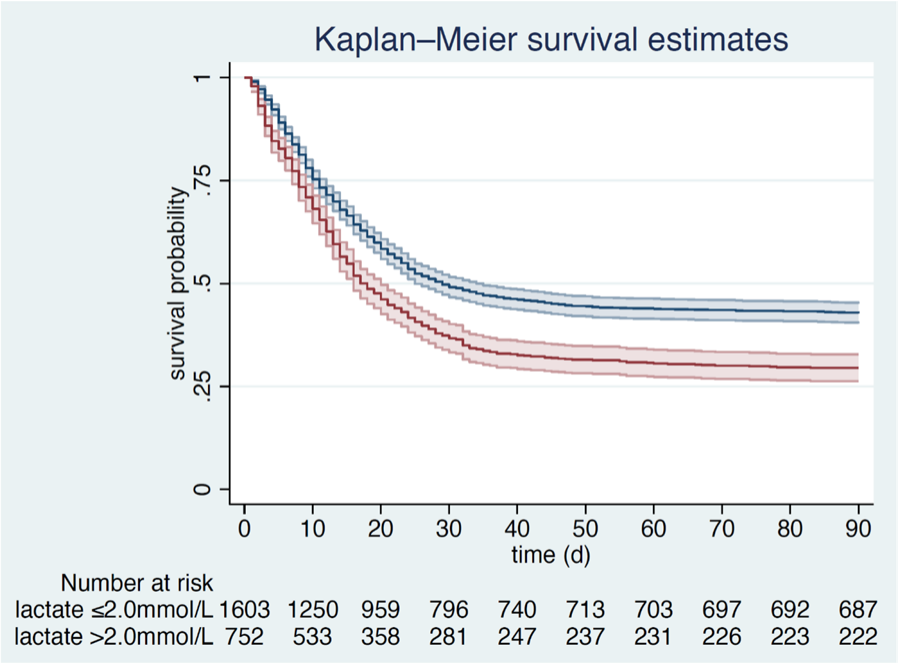
Kaplan–Meier for patients with a baseline lactate ≥ 2 mmol/L (red line) compared to patients with a baseline lactate < 2 mmol/L (blue line) (3-month mortality, ± standard deviation)

In the multivariable analysis, the maximum lactate concentration on day 1 was independently associated with ICU mortality (aOR 1.06 95% CI 1.02–1.11; *p* = 0.007), 30-day mortality (aOR 1.07 95% CI 1.02–1.13; *p* = 0.005) and 3-month mortality (aOR 1.15 95% CI 1.08–1.24; *p* < 0.001) after adjustment for age, SOFA, CFS and sex (Fig. 3).

### ∆ Serum lactate in 24 h

**Fig. 4:**
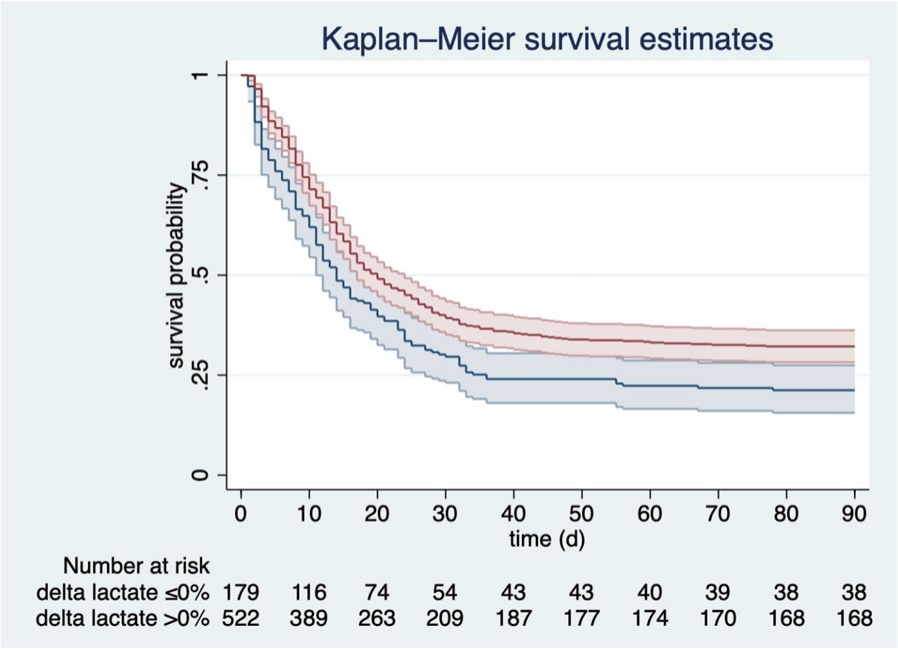
Kaplan–Meier for patients with a ∆ lactate 24 h > 0% (red line) compared to patients with a ∆ lactate 24 h ≤ 0% (blue line) (3-month mortality, ± standard deviation)

In 826 patients (29%), there was a baseline lactate ≥ 2 mmol/L and sufficient data to calculate the ∆ serum lactate over 24 h. In both sub-groups, patients were predominantly male (*p* = 0.37, Table 3). There were no differences regarding age, SOFA score on admission, prior hospitalizations, or the duration of symptoms before ICU admission. The median CFS did not differ (Table 3). For pre-existing comorbidities, there was no difference between groups except for pulmonary diseases, which were significantly more common in those patients with rising lactate [27% (*n* = 58) vs. 19% (*n* = 117), *p* = 0.017]. Intensive care treatment, especially regarding non-invasive ventilation [27% (57) vs. 24% (146), *p* = 0.45], vasoactive drugs [70% (151) vs. 74% (444), *p* = 0.34], and renal replacement therapy [21% (45) vs. 18% (112), *p* = 0.42] did not differ between both groups. In patients with ∆ serum lactate over 24 h less than 0, intubation occurred in 71% (152) compared to 76% (464) (*p* = 0.12), prone positioning was used in 48% (72) compared to 49% (226) (*p* = 0.76). During the ICU stay, 40 patients with a ∆ serum lactate over 24 h less than 0 received a tracheostomy (19%), while this was true for 109 patients from the group of patients with a ∆ serum lactate ≥ 0% (18%, *p* = 0.79). Therapy limitations or de-escalations did also not differ between groups. Therapy was withheld in 29% (62) of the patients with a negative ∆ serum lactate, and in 30% (185) of the other group (*p* = 0.68), while treatment was withdrawn in 19% (40) and 22% (134) of the patients, respectively (*p* = 0.32). Length of stay was longer in patients with a ∆ Lactate 24 h > 0% [285 (439) h vs. 348 h (400), p = 0.029]. After exclusion of non-survivors, there was no significant difference between both groups [475 (526) h vs. 456 h (552), *p* = 4.77]. The duration of mechanical ventilation was significantly longer in patients with a positive ∆ Lactate 24 h [210 h (390) vs. 277 (340), *p* = 0.106]. However, after exclusion of non-survivors, the duration of mechanical ventilation was comparable [323 h (414) vs. 307 h (436) *p* = 0.364]. ICU mortality was significantly higher for patients with rising lactate [61% (*n* = 131) vs. 50% (*n* = 303), *p* = 0.004, Table 4, Fig. 4].

**Table 3:** Baseline characteristics of the subgroup analysis according to the Δ Lactate 24 h [%]

Variables	∆ Lactate 24 h ≤ 0%	∆ Lactate 24 h > 0%	*p* value
	*n* = 215 (26%)	*n* = 611 (74%)	
Male gender [*n*] (%)	152 (71%)	447 (73%)	0.37
Age [years] (IQR)	73–79 (75)	72–79 (76)	0.60
Age 70–79 [*n*] (%)	180 (80%)	484 (76%)	0.28
Age 80–89 [*n*] (%)	42 (19%)	143 (23%)	0.22
Age > 90 [*n*] (%)	3 (1%)	6 (1%)	0.63
CFS (IQR)	2–4 (3)	2–4 (3)	0.13
Diabetes [*n*] (%)	75 (35%)	231 (38%)	0.43
Coronary vascular disease [*n*] (%)	46 (22%)	154 (26%)	0.23
Chronic renal failure [*n*] (%)	43 (20%)	102 (17%)	0.27
Arterial hypertension [*n*] (%)	140 (65%)	403 (66%)	0.84
Pulmonary disease [*n*] (%)	58 (27%)	117 (19%)	0.017
Heart failure [*n*] (%)	42 (20%)	108 (18%)	0.85
SOFA score (IQR)	4–9 (6)	4–8 (6)	0.63

*CFS* clinical frailty scale, *SOFA* score sequential organ failure assessment for the first 24 h, *IQR* interquartile range

**Table 4:** Primary and secondary outcomes of the subgroup analysis according to the Δ Lactate 24 h [%]

Variables	∆ Lactate 24 h ≤ 0%	∆ Lactate 24 h > 0%	*p* value
	*n* = 225 (26%)	*n* = 634 (74%)	
ICU mortality	131 (61%)	303 (50%)	0.004
30-day mortality	128 (62%)	331 (55%)	0.077
3-month mortality	139 (79%)	353 (68%)	0.007
ICU length of stay (hours)	285 (439)	348 (400)	0.029
Duration of mechanical ventilation (hours)	210 (390)	277 (340)	0.106

*ICU* intensive care unit

Although not statistically significant, ∆ lactate was associated with ICU mortality after multivariable adjustment for age, SOFA score, and gender (aOR 0.997 95% CI 0.993–1.000; *p* = 0.05). A decreasing lactate (∆ lactate > 0%) was inversely associated with ICU mortality (OR 0.63 95% CI 0.46–0.87; *p* = 0.004) and remained so after multivariable adjustment for SOFA score, age, CFS and gender (aOR 0.60 95% CI 0.42–0.85; *p* = 0.004).

## Discussion

This sub-group analysis in very old ICU patients with COVID-19 examined the value of baseline lactate and lactate kinetics as predictors of outcome. Both patients with elevated baseline lactate and patients with rising lactate suffered from significantly higher ICU-mortality. These parameters were independently associated with ICU-mortality. To our knowledge, this study represents the first prospective observational study in critically ill old patients with COVID-19 on the value of lactate and lactate kinetics for outcome prediction.

These findings are of pivotal importance as lactate and its kinetics are already amongst the most important laboratory parameters for diagnosing septic shock and guiding treatment. The current sepsis guidelines advocate measurement of lactate at ICU admission and during ICU stay as one of the best variables to assess the response to treatment [14]. Indeed, a meta-analysis by Pan et al. included seven randomized controlled trials with 1301 patients to compare the early lactate clearance-directed therapy to central venous oxygen saturation (ScvO_2_)-guided therapy as a potentially more effective resuscitation target. They concluded that the use of an early lactate clearance-directed therapy resulted in a decreased in-hospital mortality, shorter ICU stay, shorter mechanical ventilation time, and lower APACHE II scores [15].

COVID-19 might be considered as a very unique type of sepsis. Thus, the “First Update on the Surviving Sepsis Campaign Guidelines on the Management of Adults with Coronavirus Disease 2019 (COVID-19) in the ICU” were developed [9]. In this statement, the authors suggest using dynamic parameters, such as serum lactate measurement, over static parameters to assess fluid responsiveness in adults with COVID-19 and shock, though with a weak level of evidence [9].

Severe COVID-19 is usually characterized by a fast-developing pneumonitis and respiratory failure. Currently, there are only very limited data about serum lactate values in this special type of pneumonitis and in pneumonia in general: Gwak et al. collected consecutive data from 397 patients who were hospitalized with community acquired pneumonia (CAP); 18% of these patients were admitted to the ICU. They found an independent association between the initial serum lactate concentration and in-hospital mortality (aOR1.24; 95% CI 1.01–1.53) [16]. A subgroup analysis of the INFAUCI-Study by Pereira et al. investigated prognostic markers in patients suffering from severe community (in most cases bacterial) acquired pneumonia. In their analysis, the mean serum lactate on admission was higher than in our study (3.0 ± 3.1 mmol/L) and independently associated with intra-hospital mortality (OR 1.13; 95% CI 1.00–1.32, in a sub-group analysis of patients with septic shock: OR 1.11; 95% CI 1.00–1.37, respectively). Interestingly, they found no association with mortality at 6 months [17]. In another retrospective analysis involving 553 patients, Jo et al. showed the non-inferiority of a baseline serum lactate combined with an early warning score compared to established pneumonia scores such as CURB-65 in predicting outcomes of patients with CAP [18].

Regarding serum lactate levels in COVID-19, the present study is in line with Goodall et al., who found a relationship between higher lactate levels (aHR 2.67) and an increased mortality in 981 patients [19]. Kayina et al. examined 235 patients and demonstrated that non-survivors had a higher baseline serum lactate (*p* < 0.01, *n* = 122) [20]. In a very small cohort of 45 ICU-patients suffering from COVID-19, Vassiliou et al. found that maximum lactate on admission was independently related to 28-day ICU-mortality. Lactate’s area under the curve for detecting 28-day ICU mortality was 0.77 (*p* = 0.008). Mixed model analysis showed that mean daily lactate levels were higher in non-survivors (*p* < 0.0001). Interestingly, when lactate levels were compared to the SOFA scores they showed a similar time pattern [21]. A retrospective cohort study by Gregoriano et al. included 99 patients with severe COVID-19. In this cohort, lactate on admission was amongst the highest prognostic factors for severe COVID-19 progression (lactate on ambient air AUC 0.67; lactate with O2 supply AUC 0.70) [22].

However, the present study also reveals another very relevant insight into old ICU patients with COVID-19: almost 70% of patients had no elevated lactate at all on admission. This contradicts previous studies, such as by Li et al. [23]. These found, in 204 older patients (≥ 60 years) diagnosed with COVID-19, an elevated lactate (median 2.3 mmol/L) in 84% of the patients [23].

## Limitations

Our study has some methodological limitations. We lack a control group of younger COVID-19 patients for comparison or a comparable age cohort of patients who were not or could not be admitted to the ICU. In addition, the COVIP database does not capture information on time from symptoms onset to ICU admission, from pre-ICU care and triage or from and level of care, while in the ICU (e.g., nurse-to-patient ratio). These treatment limitations may affect the care of older ICU patients [24]. Participating countries varied widely in their care structure. This results in a large heterogeneity of treatments. The study also cannot answer whether lactate kinetics-guided therapy prospectively gives a mortality benefit in critically ill septic patients with COVID-19. When lactate values were documented, only the first 48 h were recorded, with the highest value per 24 h in each case. However, these parameters have been used as a benchmark in most studies to date, so the determination seems more than adequate to answer the hypothesis. The ∆ Lactate subgroup analysis lacks patients who died in the first 24 h. However, this accounted only for 32 patients of the study cohort. Pre-existing liver disease might influence lactate and its kinetics. Nevertheless, in the multivariate analysis, results have been adjusted to SOFA score which includes liver function. Possibly, patients with pulmonary artery embolism are more frequently in shock leading to elevated lactate. Though, this association is speculative as the study did not investigate the occurrence of pulmonary artery embolism as there were no reports of clustered pulmonary artery emboli when establishing the COVIP study design in February 2020.

## Conclusion

In critically ill old intensive care patients suffering from COVID-19, most critically ill old COVID-19 patients had normal serum lactate on admission. However, in those who had an elevated lactate, lactate and lactate kinetics are valuable tools for outcome prediction.

## Supplementary Information

**Additional file 1.** List of Collaborators: COVIP-Study.

## Data Availability

Individual participant data that underlie the results reported in this article are available to investigators whose proposed use of the data has been approved by the COVIP steering committee. The anonymized data can be requested from the authors if required.
