# Supplementary material for: Lactate is associated with mortality in very old intensive care patients suffering from COVID-19: results from an international observational study of 2860 patients
Source: Ann Intensive Care. 2021 Aug 21;11:128. doi: 10.1186/s13613-021-00911-8 (PMC8379577; doi:10.1186/s13613-021-00911-8)
Supplement: Supplementary file 1 — Additional file 1. List of Collaborators: COVIP-Study. [file 13613_2021_911_MOESM1_ESM.docx]

**List Of Collaborators: COVIP-Study**

| Hospital | City | Name |
| --- | --- | --- |

**Austria**

| Medical University Graz | Graz | Philipp Eller |
| --- | --- | --- |
| Medical University Innsbruck | Innsbruck | Michael Joannidis |

**Belgium**

| Ziekenhuis Oost-Limburg | Genk | Dieter Mesotten |
| --- | --- | --- |
| CHR Haute Senne | Soignies | Pascal Reper |
| Ghent University Hospital | Ghent | Sandra Oeyen |
| AZ Sint-Blasius | Dendermonde | Walter Swinnen |
| Clinique Saint Pierre Ottignies | Ottignies | Nicolas Serck |
| Universitair Ziekenhuis Brussel | Brussel | Elisabeth Dewaele |

**Colombia**

| Instituto Del Corazon De Bucaramanga - Sede Bogota | Bogota | Edwin Chapeta |
| --- | --- | --- |

**Denmark**

| Herlev Og Gentofte Hospital | Herlev | Helene Brix |
| --- | --- | --- |
| Slagelse | Slagelse | Jens Brushoej |
| Regionshospitalet Horsens | Horsens | Pritpal Kumar |
| Odense University Hospital | Odense | Helene Korvenius Nedergaard |
| Sygehus Lillebælt | Kolding | Helene Korvenius Nedergaard |
| Regionshospitalet Viborg | Viborg | Tim Koch Johnsen |
| Sygehus Sønderjylland | Aabenraa | Camilla Bundesen |
| Regionshospitalet Herning | Herning | Maria Aagaard Hansen |
| Nordsjællands Hospital | Hillerød | Stine Uhrenholt |
| Regionshospitalet Randers | Randers | Helle Bundgaard |
| Aarhus University Hospital | Aarhus | Jesper Fjølner |

**England**

| Musgrove Park | Taunton | Richard Innes |
| --- | --- | --- |
| Princess Alexandra Hospital Harlow | Essex | James Gooch |
| Royal Papworth Hospital | Cambridge | Lenka Cagova |
| Royal Surrey Hospital NHS Foundation Trust | Guildford | Elizabeth Potter |
| Russells Hall | Dudley | Michael Reay |
| Tunbridge Wells Hospital | Tunbridge Wells | Miriam Davey |
| Walsall Manor Hospital | Walsall | Mohammed Abdelshafy Abusayed |
| West Suffolk NHS Foundation Trust | Bury St Edmunds | Sally Humphreys |
| Queen Elizabeth Hospital | London | Amy Collins |
| Northumbria Healthcare NHS Foundation Trust | Newcastle | Avinash Aujayeb |
| St George´S Hospital London | London | Susannah Leaver |
| University Hospital Lewisham | South London | Waqas Khaliq |

**Egypt**

| One Day Surgery | Nasr City | Ayman Abdelmawgoad Habib |
| --- | --- | --- |
| Kar Al-Ainy Cairo University Hospital | Cairo | Mohammed A Azab |
| Quweisna Central Hospital | Quweisna | Kyrillos Wassim |
| Mayo Isolation Hospital | Cairo Governorate | Yumna A. Elgazzar |
| One Day Surgery Hospital | One Day Surgery Hospital | Rehab Salah |
| Mostafa Mahmoud Specialized Hospital | Cairo | Hazem Maarouf Abosheaishaa |
| Assiut University Hospital | Assiut | Aliae AR Mohamed Hussein |
| Alazhar University Hospitals | Cairo | Ahmed Y. Azzam |
| Mansoura University Hospital | Mansoura | Samar Tharwat |
| Minia University Hospitals | Minia | Yasmin Khairy Nasreldin Mohamed Ali |
| Wingat Royal Hospital | Alexandria | Omar Elmandouh |
| Aswan University Hospital | Aswan | Islam Galal |
| Assiut University Hospital | Assiut | Ahmed Abu-Elfatth |
| Alexandria University Hospital | Alexandria | Karam Motawea |
| Alexandria Main University Hospital | Alexandria | Mohammad Elbahnasawy |
| Alexandria Main University Hospital | Alexandria | Mostafa Shehata |
| TANTA UNIVERSITY Emergency Hospital | TANTA | Mohamed Elbahnasawy |
| Alexandria Main University Hospital | Alexandria | Mostafa Tayeb |
| Medical Research Institute | Alexandria | Nermin Osman |
| Kafr Elsheikh University Hospital | Kafr Elsheikh | Wafaa Abdel-Elsalam |
| Assiut University Hospital | Assiut | Aliae Mohamed Hussein |
| Nasr City Hospital For Health Insurance | Cairo | Amer Aldhalia |

**France**

| Hôpital Privé Claude Galien | Quincy Sous Sénart | Arnaud Galbois |
| --- | --- | --- |
| Saint Antoine | Paris | Bertrand Guidet |
| Hôpital Ambroise Paré | Boulogne Billancourt | Cyril Charron |
| Hopital Européen Georges Pompidou | Paris | Caroline Hauw Berlemont |
| CHU De Besançon | Besançon | Guillaume Besch |
| Dieppe General Hospital | Dieppe | Jean-Philippe Rigaud |
| CHU Amiens | Amiens | Julien Maizel |
| Tenon | Paris | Michel Djibré |
| Clinique Du Millenaire | Montpellier | Philippe Burtin |
| Marne La Vallee | Jossigny | Pierre Garcon |
| CHU Lille | Lille | Saad Nseir |
| CHU De Caen | Caen | Xavier Valette |
| Compiegne Noyon Hospital | Compiegne | Nica Alexandru |
| Cochin | Paris | Nathalie Marin |
| CH Pau | Pau | Marie Vaissiere |
| Victor Dupouy | Argenteuil | Gaëtan Plantefeve |
| Victor Dupouy | Argenteuil | Hervé Mentec |
| CH Saint Philibert | Lomme Lez Lille | Thierry Vanderlinden |
| Beaujon | Clichy | Igor Jurcisin |
| Lariboisière | Paris | Buno Megarbane |
| Lariboisière | Paris | Benjamin Glenn Chousterman |
| Saint-Louis | Paris | François Dépret |
| Saint Antoine | Paris | Marc Garnier |
| Louis Mourier | Colombes | Sebastien Besset |
| Avicenne | Bobigny | Johanna Oziel |
| Centre Hospitalier De Versailles | Le Chesnay | Alexis Ferre |
| Robert Debré | Paris | Stéphane Dauger |
| Saint-Louis | Paris | Guillaume Dumas |
| Sainte-Anne | Paris | Bruno Goncalves |
| CHU De Besancon | Besançon | Lucie Vettoretti |
| CH Dr SCHAFFNER, Reanimation Polyvalente | Lens | Didier Thevenin |

**Germany**

| Charité - Universitätsmedizin Berlin | Berlin | Stefan Schaller |
| --- | --- | --- |
| Florence-Nightingale-Krankenhaus | Duesseldorf | Muhammed Kurt |
| Kliniken Nordoberpfalz AG Klinikum Weiden | Weiden | Andreas Faltlhauser |
| Evangelisches Krankenhaus Düsseldorf | Düsseldorf | Christian Meyer |
| Malteser Krankenhaus St. Franziskus Hospital | Flensburg | Milena Milovanovic |
| Uniklinik Schleswig-Holstein Campus Kiel | Kiel | Matthias Lutz |
| Johanna-Etienne-Krankenhaus | Neuss | Gonxhe Shala |
| Kliniken Maria Hilf | Mönchengladbach | Hendrik Haake |
| Krankenhaus Bethanien GmbH Solingen | Solingen | Winfried Randerath |
| Uniklinik Düsseldorf | Düsseldorf | Anselm Kunstein |
| University Hospital Würzburg | Würzburg | Patrick Meybohm |
| St Vincenz | Limburg | Stephan Steiner |
| University Hospital Ulm | Ulm | Eberhard Barth |
| Marienhospital Aachen | Aachen | Tudor Poerner |
| University Hospital Leipzig / Klinik Und Poliklinik Für Anästhesiologie Und Intensivtherapie | Leipzig | Philipp Simon |
| Charité - Universitätsmedizin Berlin | Berlin | Marco Lorenz |
| Städtische Kliniken Mönchengladbach | Mönchengladbach | Zouhir Dindane |
| Charité - Universitätsmedizin Berlin | Berlin | Karl Friedrich Kuhn |
| Klinikum Darmstadt Gmbh | Darmstadt | Martin Welte |
| Elisabeth-Krankenhaus Essen | Essen | Ingo Voigt |
| Klinikum Konstanz | Konstanz | Hans-Joachim Kabitz |
| Medical Center - University Of Freiburg | Freiburg | Jakob Wollborn |
| St. Franziskus-Hospital Münster | Münster | Ulrich Goebel |
| University Hospital Cologne | Cologne | Sandra Emily Stoll |
| University Hospital Duesseldorf | Duesseldorf | Detlef Kindgen-Milles |
| Essen University Hospital | Essen | Simon Dubler |
| University Hospital Duesseldorf | Düsseldorf | Christian Jung |
| Klinikum Rechts der Isar, Technical University | Munich | Kristina Fuest |
| Universitätsmedizin der Johannes-Gutenberg-Universität Mainz | Mainz | Michael Schuster |
| St Vincenz | Limburg | Stephan Steiner |

**Greece**

| General Hospital of Larissa | LARISSA | Antonios Papadogoulas |
| --- | --- | --- |
| General University Hospital Of Patras | Patras | Francesk Mulita |
| Sotiria Hospital National And Kapodistrian University Of Athens | Athens | Nikoletta Rovina |
| Ught Ahepa | Thessaloniki | Zoi Aidoni |
| University Hospital (Attikon) | Haidari | Evangelia Chrisanthopoulou |
| University Hospital of Heraklion | Heraklion | Eumorfia Kondili |
| University Hospital of Ioannina | Ioannina | Ioannis Andrianopoulos |

**India**

| Sanjay Gandhi Postgraduate Institute Of Medical Sciences (SGPGIMS) | Lucknow | Mohan Gurjar |
| --- | --- | --- |

**Iran**

| Imam Reza | Tabriz | Ata Mahmoodpoor |
| --- | --- | --- |

**Iraq**

| Zafaraniyah General Hospital | Baghdad | Rand Hussein |
| --- | --- | --- |
| Al-Amal Hospital In Najaf | Al-Najaf | Maytham Aqeel Al-Juaifari |
| Ibn Zuhur Hospital | Baghdad | Abdullah Khudhur Ahmed Karantenachy |

**Israel**

| Hadassah University Medical Center | Jerusalem | Sigal Sviri |
| --- | --- | --- |

**Ireland**

| Cork University Hospital | Cork | Ahmed Elsaka |
| --- | --- | --- |
| Mater Misericordiae University Hospital | Dublin | Brian Marsh |

**Italy**

| Policlinico S. Orsola-Malpighi | Bologna | Vittoria Comellini |
| --- | --- | --- |

**Jordan**

| Al-Esraa Hospital | Amman | Farah Al-Ali |
| --- | --- | --- |
| King Hussein Medical Center | Amman | Sari Almani |
| Irbid Speciality Hospital | Irbid | Almu´Atasim Khamees |
| Jordan University Hospital | Amman | Khayry Al-Shami |

**Lebanon**

| Nini | Tripoli | Ibrahim Salah El Din |
| --- | --- | --- |

**Libya**

| Benghazi Medical Center | Bengazi | Taha Abubaker |
| --- | --- | --- |
| Tripoli University Hospital | Tripoli | Hazem Ahmed |
| Askar | Suq Elkamis | Ahmed Rabha |
| Al-Zawia Isolation Hospital | Al-Zawia | Abdulmueti Alhadi |
| Misurata Medical Center | Misurata | Marwa Emhamed |
| Sebha Medical Center | Sebha | Saedah Abdeewi |
| Almwasfat Hospital | Tripoli | Abdurraouf Abusalama |
| Sorman Teaching Hospital | Sorman | Abdulmueti Alhadi |
| Alshahid Attia Alkasah General Hospital | Alkufra | Mohammed Huwaysh |
| Ghadames Central Hospital | Ghadames | Esraa Abdalqader Alghati |

**Morocco**

| CHU Ibn Sina De Rabat | Rabat | Abdelilah Ghannam |
| --- | --- | --- |

**Mexico**

| Instituto Nacional De Ciencias Medicas Y Nutricion Salvador Zubiran | Mexico City | Silvio A Namendys-Sylva |
| --- | --- | --- |

**Netherland**

| Alrijne Zorggroep | Leiderdorp | Martijn Groenendijk |
| --- | --- | --- |
| Canisius Wilhelmina Hospital | Nijmegen | Mirjam Evers |
| Canisius Wilhelmlina Ziekenhuis | Nijmegen | Mirjam Evers |
| Diakonessenhuis Utrecht | Utrecht | Lenneke Van Lelyveld-Haas |
| Haga Ziekenhuis | The Hague | Iwan Meynaar |
| Medisch Spectrum Twente | Enschede | Alexander Daniel Cornet |
| Radboudumc | Nijmegen | Marieke Zegers |
| University Medical Center Groningen | Groningen | Willem Dieperink |
| University Medical Center Utrecht | Utrecht | Dylan De Lange |
| Zuyderland Mc | Heerlen | Tom Dormans |

**Norway**

| Haugesund Hospital | Haugesund | Michael Hahn |
| --- | --- | --- |
| Haukeland University Hospital | Bergen | Britt Sjøbøe |
| Kristiansund Hospital Helse Møre Og Romsdal HF | Kristiansund | Hans Frank Strietzel |
| Oslo University Hospital | Oslo | Theresa Olasveengen |
| Oslo University Hospital Rikshospitalet Medical | Oslo | Luis Romundstad |
| Ålesund Hospital | Ålesund | Finn H. Andersen |

**Oman**

| Khoula Hospital | Muscat | John George Grace Massoud |
| --- | --- | --- |

**Pakistan**

| MTI Lady Reading Hospital | Peshawar | Aamir Ghafoor Khan |
| --- | --- | --- |

**Palestine**

| Dura Hospital | Hebron | Shahd Al-Qasrawi |
| --- | --- | --- |
| Alia Governmental Hospital | Hebron | Sarah Amro |

**Poland**

| Clinical Hospital Heliodor Święcicki Medical University of Karol Marcinkowski In Poznan | Poznan | Anna Kluzik |
| --- | --- | --- |
| Infant Jesus Clinical Hospital Medical University of Warsaw | Warsaw | Paweł Zatorski |
| Jagiellonia University Hospital Cracow | Cracow | Tomasz Drygalski |
| Military Hospital | Krakow | Wojciech Szczeklik |
| Military Institute Of Medicine | Warsaw | Jakub Klimkiewicz |
| Pomeranian Medical University | Szczecin | Joanna Solek-Pastuszka |
| Provincial Specialist Hospital | Olsztyn | Dariusz Onichimowski |
| SPSK-1 | Lublin | Miroslaw Czuczwar |
| University Hospital In Opole | Opole | Ryszard Gawda |
| Uniwersyteckie Centrum Kliniczne W Gdańsku | Gdańsk | Jan Stefaniak |
| Voivodship Hospital In Poznan | Poznan | Karina Stefanska-Wronka |
| ZDROWIE Sp. Z O.O. | Kwidzyn | Ewa Zabul |

**Portugal**

| Centro Hospitalar De Tondela-Viseu EPE | Viseu | Ana Isabel Pinho Oliveira |
| --- | --- | --- |
| Centro Hospitalar Do Médio Tejo | Abrantes | Rui Assis |
| Centro Hospitalar E Universitário São João | Porto | Maria De Lurdes Campos Santos |
| Centro Hospitalar Trás-Os-Montes E Alto Dour | Vila Real | Henrique Santos |
| Hospital de Curry, Centro Hospital Universitário de Lisboa Central | Lisbon | Filipe Sousa Cardoso |
| Hospital De Beatriz Ângelo | Loures | André Gordinho |

**Romania**

| Clinical Emergency Hospital Bucharest | Bucharest | Ioana Marina Grintescu |
| --- | --- | --- |
| Fundeni Clinical Institute | Bucharest | Dana Tomescu |

**Saudi Arabia**

| International Medical Center | Jeddah | Mohamed Raafat Badawy |
| --- | --- | --- |

**Spain**

| Clínico Universitario Lozano-Blesa | Zaragoza | M José Arche Banzo |
| --- | --- | --- |
| Clínico Universitario Lozano-Blesa | Zaragoza | Begoña Zalba-Etayo |
| Complejo Asistencial De Segovia | Segovia | Patricia Jimeno Cubero |
| Complexo Hospitalario Universitario Ourense | Ourense | Jesús Priego |
| Corporació Sanitària Parc Taulí | Sabadell | Gemma Gomà |
| Germans Trias I Pujol | Badalona | Teresa Maria Tomasa-Irriguible |
| H. Universitari I Politècnic La Fe | Valencia | Susana Sancho |
| Hospital Alvaro Cunqueiro | Vigo | Aida Fernández Ferreira |
| Hospital De Tortosa Verge De La Cinta | Tortosa | Eric Mayor Vázquez |
| Hospital General Universitario De Albacete | Albacete | Ángela Prado Mira |
| Hospital Universitari Sagrat Cor | Barcelona | Mercedes Ibarz |
| Hospital Universitario De Burgos | Burgos | David Iglesias |
| Hospital Universitario De Getafe | Getafe | Susana Arias-Rivera |
| Hospital Universitario De Getafe | Getafe | Fernando Frutos-Vivar |
| Hospital Universitario Rey Juan Carlos | Mostoles | Sonia Lopez-Cuenca |
| Hospital Universitario Rio Hortega | Valladolid | Cesar Aldecoa |
| Hospital Universitario Río Hortega | Valladolid | David Perez-Torres |
| Hospital Universitario Río Hortega | Valladolid | Isabel Canas-Perez |
| Hospital Universitario Río Hortega | Valladolid | Luis Tamayo-Lomas |
| Hospital Universitario Río Hortega | Valladolid | Cristina Diaz-Rodriguez |
| Miguel Servet University Hospital | Zaragoza | Pablo Ruiz De Gopegui |

**Sudan**

| Wad Medani Teaching Hospital | Wad Medani | Mahmoud Saleh |
| --- | --- | --- |
| Medical Military Hospital | Khartoum | Momin Majed Yousuf Hilles |
| Khartoum Bahri Hospital | Khartoum | Enas M. Y Abualqumboz |

**Switzerland**

| Centre Hospitalier Universitaire Vaudois | Lausanne | Nawfel Ben-Hamouda |
| --- | --- | --- |
| Clinica Luganese Moncucco | Lugano | Andrea Roberti |
| Fribourg Hospital | Fribourg | Yvan Fleury |
| Geneva University Hospitals | Geneva | Nour Abidi |
| Inselspital Bern | Bern | Joerg C. Schefold |
| Kantonspital Thurgau Frauenfeld | Frauenfeld | Ivan Chau |
| Kantonspital Thurgau Frauenfeld | Frauenfeld | Alexander Dullenkopf |

**Syria**

| Al-Mouwasat University Hospital | Damascus | Mohammad Karam Chaaban |
| --- | --- | --- |
| Al-Assad University Hospital | Damascus | Mohammed Mouaz Shebani |
| Sham | Idlib | Ahmad Hmaideh |
| Tishreen University Hospital | Lattakia | Aymen Shaher |

**Turkey**

| Kanuni Sultan Suleyman Education and Training Hospital | Istanbul | Ayca Sultan Sahin |
| --- | --- | --- |
| Kartal Dr. Lutfi Kirdar Training and Research Hospita | Istanbul | Kemal Tolga Saracoglu |

**USA**

| SUNY Downstate | Brooklyn | Mohammed Al-Sadawi |
| --- | --- | --- |

**Wales**

| Glan Clwyd Hospital | Bodelwyddan | Richard Pugh |
| --- | --- | --- |
| Wrexham Maelor Hospital | Wrexham | Sara Smuts |

**Yemen**

| Zayad Hospital | Sana'a City | Rafat Ameen Mohammed Al-Saban |
| --- | --- | --- |
